# Determination of the Molar Fraction and Enantiomeric Excess of Electrosprayed Amino Acid Anions Employing Photoelectron Circular Dichroism

**DOI:** 10.1021/acs.analchem.4c05964

**Published:** 2025-02-19

**Authors:** Jon Henrik Both, Anastasiya Beliakouskaya, Karl-Michael Weitzel

**Affiliations:** Chemistry Department, Philipps Universität Marburg, Marburg 35032 Germany

## Abstract

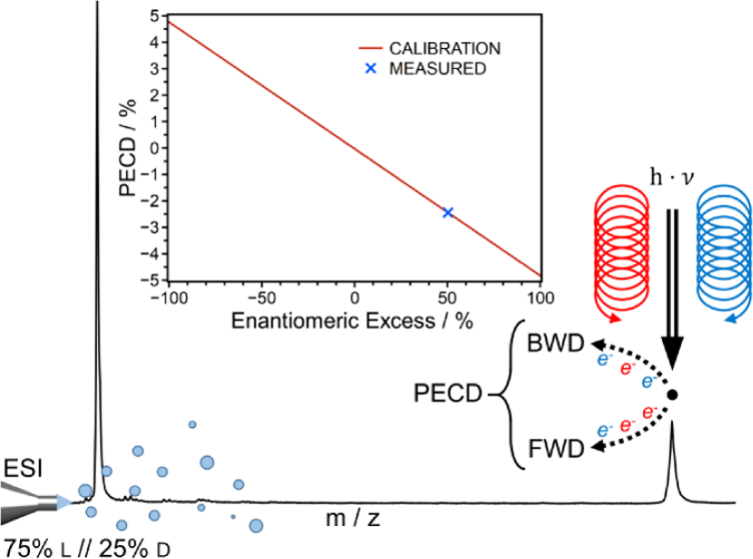

The quantification of molar fractions and enantiomeric excess has been demonstrated in mixtures of d- and l-tryptophan and d- and l-phenylalanine, respectively, avoiding derivatization of the analyte with additional reagents or separation steps. The technique is based on electrospray ionization (ESI), which allows the generation of anions of nonvolatile compounds such as amino acids or large biomolecules. Electrons are photodetached from these anions. The distribution of forward and backward scattered photoelectrons is analyzed, leading to photoelectron circular dichroism (PECD), the observable of interest. The quantification of the concept is proven by blind measurements analyzing mixtures of unknown composition. The quantification of enantiomeric excess (ee) values is not only possible for signals originating from the molecular anion but also for the molecular dimer anion. The ESI-PECD technique is known to be applicable to large chemical entities of several thousand Daltons.

## Introduction

Most biological systems, including amino acids, proteins, sugars, and nucleotides, are chiral. The interaction of biological systems with chiral molecules, e.g., pharmaceuticals, also depends on the handedness of such chiral compounds. As a consequence, knowledge of molecular chirality and, even more, the enantiomeric purity is very important for the application of new substances in biological and medical contexts.^1−3^ Especially in recent years, pharmacological and medical research has strengthened the focus on enantiomerically pure substances to reduce the side effects of administered drugs.^4,5^ Therefore, reliable methods for the identification and quantification of enantiomeric molecules are also needed and have been the subject of extensive research.^3,6^

Our recent study focuses on the application of the PECD of anions for the analysis of the molar fraction and the ee, respectively. Currently, available methods for the analysis of the molar fraction of enantiomers, like chiral high-performance liquid chromatography (chiral HPLC),^7^ electronic CD spectroscopy (ECD),^8^ fluorescence-detected CD,^9^ or vibrational CD (VCD)^10,11^ spectroscopy, require time-consuming separation steps or high analyte concentrations due to low signal-to-noise ratios. Nuclear magnetic resonance approaches to ee determination in general involve interaction with chiral auxiliaries in solution.^12−15^ The signal-to-noise ratio issue can be resolved using stoichiometric derivatization steps like covalent functionalization,^16−18^ metal-complexation,^18−22^ or macrocyclic host molecules.^23−26^ However, all of these methods need at least one additional chemical reaction step that possibly is analyte-specific and lengthens the analysis. The role of separation in the analysis of chiral compounds has recently been reviewed.^27^ New methods based solely on mass spectrometry are currently under investigation to fully utilize the versatility and speed of mass spectrometers.^28−30^

To circumvent the low signal-to-noise ratio of the CD spectroscopy and, therefore, eliminate the need for derivatization, we have chosen the angular distribution of photoelectrons in the form of PECD as a suitable parameter for our analysis. PECD is obtained from a measurement of the yield of electrons ejected from an analyte of interest in the forward or backward direction relative to the propagation of the light beam causing the electron ejection. The ejection of an electron from a neutral analyte constitutes conventional photoionization. Ejection of an electron from an anion is termed photodetachment and leads to a neutral molecule and a free electron.

The PECD effect is already well-studied for the case of the photoionization of neutral, chiral analytes and shows a significantly higher effect than ECD.^31,32^ The first experiments analyzing the enantiomeric excess using PECD have already been reported for neutral analytes.^33−35^ These experiments have recently been extended to the measurement and analysis of photoelectron elliptical dichroism (PEELD).^36−38^ The focus of this work is on extending the PECD method for ee determination in combination with an electrospray ion source, providing access to analytes with low vapor pressure. The determination of electronic circular dichroism of ESI-sprayed anionic DNA fragments was first reported by Daly et al.^39^ The measurement of PECD in the photodetachment from electrosprayed anions was originally reported by Krüger and Weitzel,^40^ and subsequently applied to large bioorganic systems.^41^ More recently, the prospect of mass selection prior to photodetachment has been elaborated.^42^ In the current work, the analysis of mass-selective PECD information will also be addressed.

In the current work, phenylalanine (Phe) and tryptophan (Trp) have been chosen as analytes for ee quantification. Both systems have already been investigated employing multiphoton ionization PECD or multiphoton ionization PEELD.^37,43^

## Experimental Section

The ESI-PECD setup for our experiments was described by Krüger and Weitzel.^40^ The light source was changed to a Radiant Dyes NarrowScan dye laser, which was operated at 234 nm using Coumarin 102, which was purchased from Radiant Dyes. The laser system was operated at 10 Hz with pulse energies ranging from 75 μJ to 100 μJ. For each measured PECD value, 300 000–400 000 laser shots were accumulated. The polarization of our laser beam was cleaned using a Glan-Taylor polarizer from Topag Lasertechnik. The circular polarization was generated using an achromatic quarter waveplate from Bernhard Halle Nachfl., yielding fractions of circular polarization of 98% (left circular polarization, LCP) and 99% (right circular polarization, RCP). We use the definition of the handedness of the light viewed from the sender of the light, as proposed by Richard Feynman.^44^

As analytes, we used commercially available l-Phe (Merck KGaA), nominal purity 99%, d-Phe (Fluorochem), nominal purity 98%, l-Trp (AppliChem GmbH), nominal purity 99%, and d-Trp (Fluorochem), nominal purity 97%. All analytes were used without further purification. The chemical purity of the analytes was taken into account for the calculation of the molar fraction, and the analytes were considered enantiomerically pure. Molar fractions stated in the context of ee determination refer to the analytes only, not including any solvent. As a solvent, pure methanol (isocratic grade for HPLC) from VWR Chemicals was used. For Phe, two enantiomerically pure stock solutions were prepared with concentrations of 2 mmol/L. For Trp, two enantiomerically pure stock solutions were prepared with concentrations of 1 mmol/L. The calibration standards and test samples were prepared using the stock solutions and 1, 2, 5, and 10 mL volumetric pipettes. All solutions were prepared in 100 mL volumetric flasks.

## Results and Discussion

All photodetachment experiments described in this work have been conducted by employing photons at 234 nm. In all cases, the corresponding photon energy has been sufficient to overcome the threshold for photodetachment from the analytes of interest. No attempts were made to determine the actual threshold. The raw data of the measurements consist of one time-of-flight mass spectrum for the anions and two electron time-of-flight (e-ToF) spectra for each laser shot, measured separately. One e-ToF is measured on the forward (FWD) and one on the backward (BWD) oriented detector. The electron signal is integrated over the relevant ToF for each laser shot. The polarization-dependent PECD value is calculated by eq 1 for each laser shot, where  is the forward detected signal,  is the backward detected signal, and p is the respective polarization.^45,46^ The polarization handedness is switched every 600 laser shots.
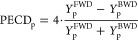
1

Ultimately, the polarization-dependent PECD values are symmetrized using eq 2.
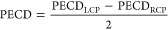
2

The uncertainty of the PECD values is calculated from the standard error (SE) of averaging the polarization-dependent PECD values using eq 3, where LCP and RCP are left and right circular polarization, respectively.
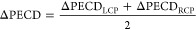
3

### Phenylalanine

**Figure 1:**
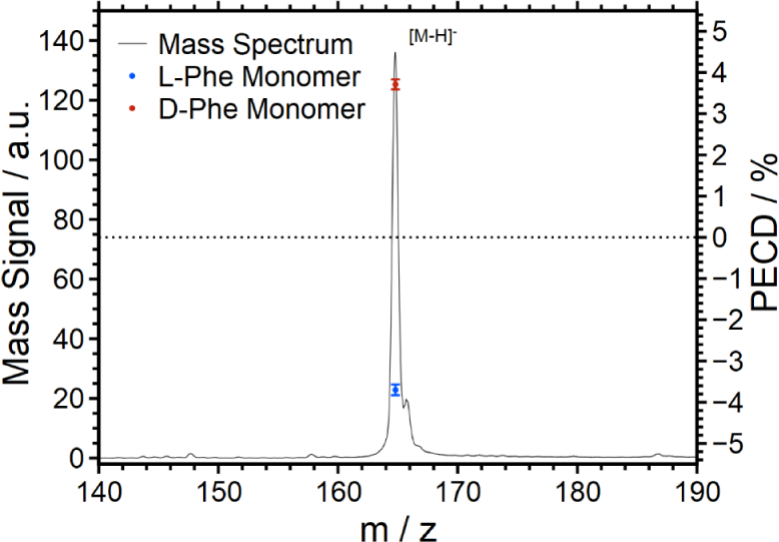
Mass spectrum of the Phe-monomer with measured PECD values.

Figure 1 shows the mass spectrum of phenylalanine in the mass region relevant to the detachment. The monomer anion is the only anion from which photodetachment is observed. The mass spectrum is measured with and without laser interaction. The difference spectrum (shown in Figure S1) indicates that photodetachment originates only from the monomer anion. The PECD measured under these conditions can therefore be assigned to the Phe-monomer. The PECD values measured, ±3.7%, are smaller than those reported by Sparling et al., who reported PECD values of −6.9% and 7.7% in their PECD study of neutral Phe.^43^ The difference in the PECD values measured is not per se surprising, since the charge state of the analyte differed and also the number of photons involved differed (multiphoton process employed by Sparling et al. versus the single photon process employed in this work). However, the values observed in this work are large enough to measure a calibration function with different molar fractions of l-Phe versus d-Phe and thus enable the ee determination.

**Figure 2:**
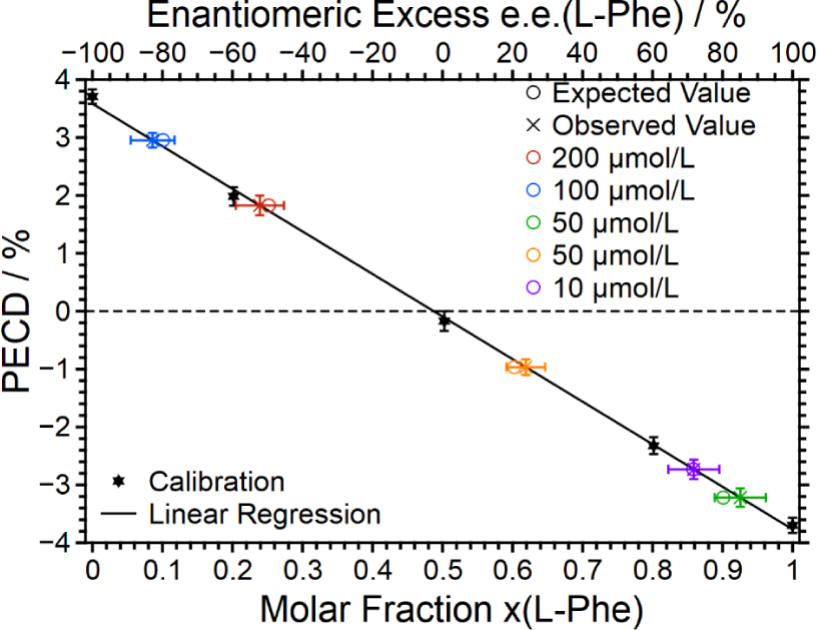
Calibration (black) for the determination of the molar fraction of Phe based on the PECD of the monomer with test measurements (colored) of different molar fractions and concentrations to validate the calibration.

To set up a calibration standard for a blind ee determination, the PECD value was measured for a total of five different mixtures of l-Phe and d-Phe, i.e., *x* = 0, 0.2, 0.5, 0.8, and 1. The measured data and the regression line are shown in Figure 2. Evidently, the PECD data scale linearly with the molar fraction and the enantiomeric excess. All data, including the uncertainty, are also listed in Table 1. The margin of error of the measurement is constant over the entire calibration range, mainly depending on the total measurement time for each standard.

**Table 1:** Calibration Standards for the Determination of the Molar Fraction of Phe with the Measured PECD Values and the Standard Error of the PECD Measurement

Calibration Standard	Concentration *c*/μmol/L	Molar Fraction *x*(l-Phe)	PECD/%	SE-PECD/%
1	50	1.00	–3.70	0.13
2	50	0.00	3.71	0.12
3	50	0.20	1.98	0.16
4	50	0.80	–2.32	0.15
5	50	0.50	–0.17	0.17

The linear regression of the calibration measurements resulted in a calibration function of the form *x*(l − Phe) = *a*· + *b* with *a* = −0.1360 ± 0.0025 and *b* = 0.4875 ± 0.0068. The coefficient of determination was calculated as *R*^2^ = 0.9990.

Subsequent to the calibration measurement, a total of five different blind samples have been analyzed. To this end, the PECD value has been measured and included along the calibration line in Figure 2 (colored crosses). For comparison, the factual composition is also indicated in Figure 2 (colored circles). Evidently, the blind determination reproduces the factual composition within the error margins. All data relevant to the blind measurement are also listed in Table 2.

**Table 2:** Test Samples with Different Molar Fractions and Concentrations, the Molar Fractions of Which Were Calculated Using the Calibration Function

Test Sample	Concentration *c*/μmol/L	*x*(l-Phe) Expected	*x*(l-Phe) Observed	PECD/%	SE-PECD/%	SE-*x*(l-Phe)
1	200	0.25	0.24	1.83	0.17	0.034
2	100	0.10	0.09	2.95	0.13	0.031
3	50	0.90	0.93	–3.22	0.16	0.037
4	50	0.60	0.62	–0.97	0.14	0.028
5	10	0.86	0.86	–2.73	0.17	0.036

It is worth noting that the blind measurements have indeed been performed for a series of concentrations in the mixtures used in the ESI process. While the calibration curve has been recorded at concentrations of 50 μmol/L, the blind measurement, in fact, tested a variation of concentrations between 10 and 200 μmol/L. One goal was to identify the smallest concentration for which an ee quantification is still possible.

Figure 2 and Table 2 demonstrate that the PECD measurement is possible down to a concentration of at least 10 μmol/L. At this concentration of 10 μmol/L, the total measurement time has to be increased in order to achieve the same uncertainty. Smaller concentrations have not been considered until now. Overall, the PECD measurement and ee determination are independent of the analyte concentration in the range indicated in Figure 2. The uncertainties of the observed molar fraction were calculated by using eq 4.
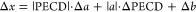
4

### Tryptophan

**Figure 3:**
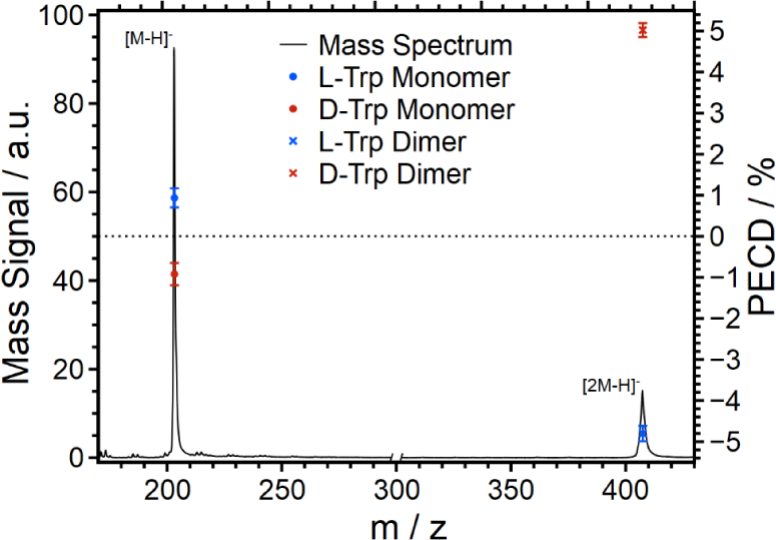
Composite mass spectrum of Trp with PECD values of the two isolated mass peaks [M – H]^−1^ and [2M – H]^−1^. Two mass spectra, optimized for the mass regions of the monomer and dimer, respectively, were recorded separately and catenated.

In order to demonstrate the versatility of the approach, we investigated a second analyte system, Trp. Figure 3 shows a composite mass spectrum of Trp. The spectrum indicates two mass regions, one for the Trp monomer and one for the Trp dimer. The two regions have been recorded with different settings of the spectrometer. For selection of the mass regions, the high-frequency voltage of the octopole was changed to preferentially transmit anions with a lower or higher mass-to-charge ratio. Furthermore, the delay between the ion pulse and the laser pulse was timed according to the mass region we wanted to detach. By adjusting the ESI settings and the laser timing, photodetachment can be induced selectively from either the monomer anion or the dimer anion of Trp. As the next step, the PECD values were measured for the pure enantiomers, i.e., l-Trp and d-Trp, for both the monomer anion and the dimer anion. The PECD data measured are also included in Figure 3 and Table 3. It is interesting to note that the PECD of the monomer anion and the dimer anion differ not only in magnitude but also in sign. While the PECD of the l-Trp monomer anion is positive, that of the l-Trp dimer anion is negative. For d-Trp, the opposite result is observed.

**Table 3:** Measured PECD Values for Trp

Enantiomer	Signal	PECD/%	SE-PECD/%
l-Trp	monomer [M – H]^−1^	0.94	0.23
	dimer [2M – H]^−1^	–4.80	0.19
d-Trp	monomer [M – H]^−1^	–0.92	0.27
	dimer [2M – H]^−1^	5.02	0.17

For Trp, Comby et al. reported PEELD data in an fs-COLTRIMS experiment.^37^ There, Trp molecules were introduced into the spectrometer by heating a target on which Trp had been deposited. This leads to rather a low analyte concentration in the gas phase and, as a consequence, a weak signal. The experiment of Comby et al. and the one presented in this work differ in the charge state of the molecules investigated, which was neutral in Comby’s work and is an anion in the current work. From an analytical point of view, the ee value of a mixture of enantiomers is affected neither by the fs-COLTRIMS experiment nor by an ESI source. Here, the ESI source has the advantage of providing access to very large bioorganic molecules. Not only are the ESI-PECD values reported above for the monomer anion larger than the corresponding numbers from Comby et al., but they are also symmetric with respect to racemates. Furthermore, the PECD observed for the Trp-dimer anions, i.e., ±5% are by far larger than the monomer values.

**Figure 4:**
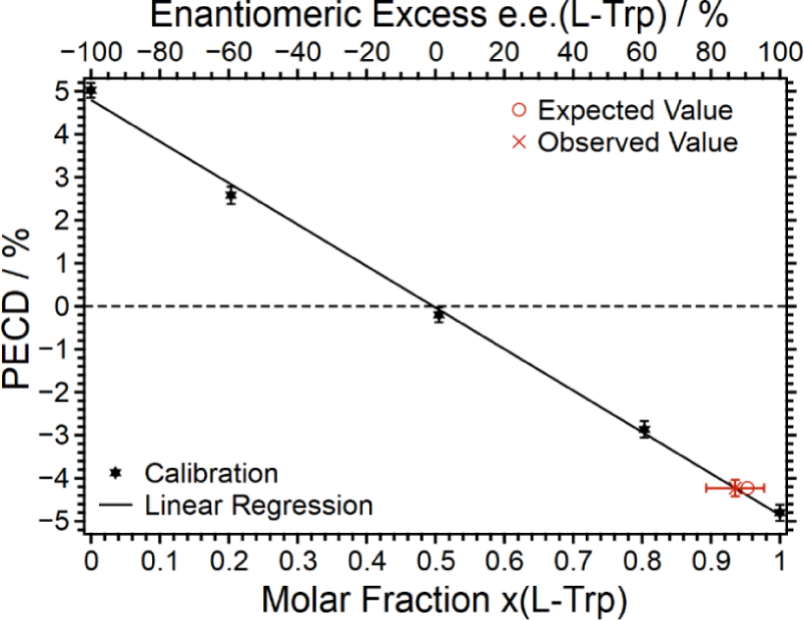
Calibration (black) for the determination of the molar fraction of Trp based on the PECD of the monoanionic dimer, with a test measurement (red) for the validation of the calibration.

At this point, the goal of this work is to demonstrate the power of ESI-PECD for the quantification of ee values. Here, the question arises of whether quantification of ee values is possible by analyzing the dimer signal PECD values, which are a factor of five larger than those of the monomer. The obvious advantage would be an improved signal-to-noise ratio. The results of these studies are presented below. Again, analogous to the Phe case described above, a calibration standard has been elaborated by measuring the PECD in the photodetachment from the dimer anion for 5 different molar fractions, i.e., *x* = 0, 0.2, 0.5, 0.8, and 1. The PECD data obtained are shown in Figure 4. Evidently, the PECD measured scales again linearly with the molar composition and ee value. The calibration data are also included in Table 4.

**Table 4:** Calibration Standards and Test Sample for the Determination of the Molar Fraction of Trp with the Measured PECD Values and Calculated Molar Fraction

Calibration Standard	Concentration *c*/μmol/L	Molar Fraction *x*(l-Trp)	PECD/%	SE-PECD/%
1	100	1.00	–4.80	0.19
2	100	0.00	5.02	0.17
3	100	0.80	–2.86	0.19
4	100	0.51	–0.20	0.18
5	100	0.20	2.58	0.20

Test Sample	Concentration *c*/μmol/L	*x*(l-Trp) Expected	*x*(l-Trp) Observed	PECD/%	SE-PECD/%	SE-*x*(l-Trp)
1	100	0.95	0.94	–4.23	0.19	0.042

The linear regression of the calibration measurements resulted in a calibration function of the form *x*(l − Trp) = *a*· + *b* with *a* = −0.1036 ± 0.0028 and *b* = 0.4969 ± 0.0100. The coefficient of determination was calculated as *R*^2^ = 0.9978.

Ultimately, once again, a blind measurement of a test sample prepared by a third person has been conducted. The measured PECD is also included in Figure 4 together with the factual composition of the test sample. The agreement is gratifying and demonstrates that indeed even the dimer signal can be exploited to quantify the ee value of an unknown enantiomeric mixture of l-Trp and d-Trp.

From an analytical point of view, the data presented above demonstrate that the PECD signal of dimers of Trp allows the quantification of ee values. The question remains whether this was to be expected, given the fact that the PECD of monomer and dimer differ in sign. The exclusive measurement of a dimer’s circular dichroism property is obviously possible in an ESI ion beam experiment. In VCD experiments in solution, the contribution of dimers to the total signal measured has been observed for concentrations larger than about 50 mmol/L.^47,48^ The VCD spectra reported for monomer and dimer conditions differed significantly, including also differences in the sign of the VCD observed. Note that the concentration of the sample solution introduced into the ESI in this work is about a factor of 500 smaller. Changes of sign for monomers and dimers of camphor have also been observed in a PECD study by Nahon et al.^49^ While the focus of the work presented here is on the PECD observed in the angular distribution of electrons, total yield CD values can also be derived from total electron yield data. However, these values were small and not well reproducible.

Given the molecules l-Trp and d-Trp conceptually, four different dimers may be discussed, i.e., the homodimers ll and dd and the heterodimers ld and dl. Quantum chemical calculations found a total of 62 different conformers (minima) for the potential energy landscape of Trp dimers, even without explicitly addressing the question of homodimers and heterodimers.^50^ Homodimers and heterodimers can, in principle, be distinguished by NMR^51^ or HPLC.^52^ The distinction between homodimers and heterodimers has also been demonstrated in ion mobility studies, ultimately even allowing ee determination^53,54^ At this point, there is no information available regarding the possible PECD value of heterodimers of Trp. The fact that the calibration curve presented in Figure 4 is linear over the entire range shown and the quantification of a blind sample is de facto possible suggests that the PECD of ld and dl heterodimers is either zero or cancels out. Clearly, a theoretical calculation of PECD of such heterodimers would help in better understanding possible contributions to a measured PECD. The conclusion that the ee determination of a blind sample is possible by analyzing the dimer signal stands as it is.

## Conclusion

We have demonstrated that the PECD of anions measured in photodetachment depends linearly on the molar fraction of the enantiomeric mixtures investigated. Ultimately, this relation allows quantification of the molar fraction and the enantiomeric excess of an unknown mixture of the enantiomers. This holds true not only for the simple monomer mass signal that was investigated for Phe but also for the dimer mass signal that was investigated in the Trp case. The fact that the measurement can be performed with different signals in the mass spectrum provides the opportunity to choose the mass signal with the highest signal-to-noise ratio and therefore possibly improves the robustness of the analysis of the molar fraction/enantiomeric excess compared to other methods that cannot differentiate between different signals of the same analyte.

We were also able to show that the measured PECD value is not dependent on the concentration of the sample. This opens the opportunity to optimize the choice of calibration standards for the best signal-to-noise ratio while not being limited to sample solutions of the given concentration. Furthermore, this allows analyzing samples with unknown concentrations without the need to analyze the concentration first. Compared to other derivatization-free methods for the analysis of the enantiomeric excess, we were able to reduce the concentration of the samples by orders of magnitude; compared to methods that rely on derivatizations of the analyte with additional reagents, our method is on par concentration-wise. The sign of the PECD values measured is different for the monomer and dimer anion. Here, the dimerization of enantiomers is considered distinctly different from derivatization by employing additional chemical entities.

The method described in this work can be realized by combining standard components of commercially available mass spectrometers and nanosecond laser systems with a simple photoelectron spectrometer, and it is hoped to find broader application in the future. The narrow bandwidth of the dye laser system used, compared to a femtosecond laser system, also allows for easy wavelength scans and identification of a wavelength where the signal-to-noise ratio is optimized for a given analytical system. From previous work, it is known that the ESI-PECD approach is applicable to peptides of several thousand Daltons, e.g., Gramicidin. Therefore, the new method provides access to sample systems that are not accessible with the methods from Kastner et al.,^34^ Comby et al.,^36,37^ and Fanood et al.,^35^ which relied solely on the vapor pressure of a given substance.

## Supporting Information Available

Mass spectra illustrating the photodetachment of Phe, Trp monomers, and dimers; raw electron time-of-flight spectra for the determination of PECD (PDF)
