# Supplementary material for: Determination of the Molar Fraction and Enantiomeric Excess of Electrosprayed Amino Acid Anions Employing Photoelectron Circular Dichroism
Source: Anal Chem. 2025 Feb 19;97(8):4499–504. doi: 10.1021/acs.analchem.4c05964 (PMC11883741; doi:10.1021/acs.analchem.4c05964)
Supplement: Supplementary file 1 — ac4c05964_si_001.pdf [file ac4c05964_si_001.pdf]

Supporting Information

Determination of the Molar Fraction and Enantiomeric Excess of Electrosprayed Amino Acid Anions Employing Photoelectron Circular Dichroism

Jon Henrik Both, Anastasiya Beliakouskaya, Karl-Michael Weitzel
Chemistry Department, Philipps Universität Marburg, 35032 Marburg, Germany

Supplementary information is provided below, regarding the difference spectra illustrating that the photodetachment is not only observed in the electron forward backward distribution discussed in the main text, but also in the mass spectra of the species under investigation. More specifically, the difference mass spectrum is the one laser on minus the one with laser off. Thus, the difference is negative.

The Figure S 1 shows the detachment of the monomer of Phe. Since we observed just the depletion of this one signal, we conclude that the PECD can be assigned to the Phe-monomer.

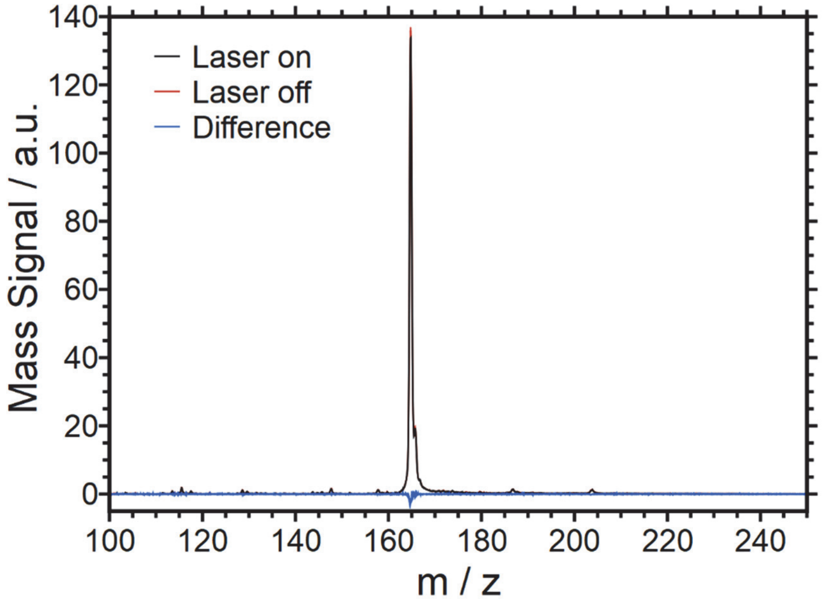

Figure S 1: Mass Spectrum of Phe. Blue: Difference induced by the laser pulse, highlighting the photodetachment of the Phe-monomer anion.

The Figure S 2 and Figure S 3 show the detachment of the monomer of Trp and the dimer of Trp independend from each other. Since we observed just the depletion of one signal each, we conclude that the PECD can be assigned to the Trp-monomer (Figure S 2) and the Trp-dimer (Figure S 3). Figure S 3 also shows a small signal at the m/z of the Trp-monomer, but a depletion is not visible.

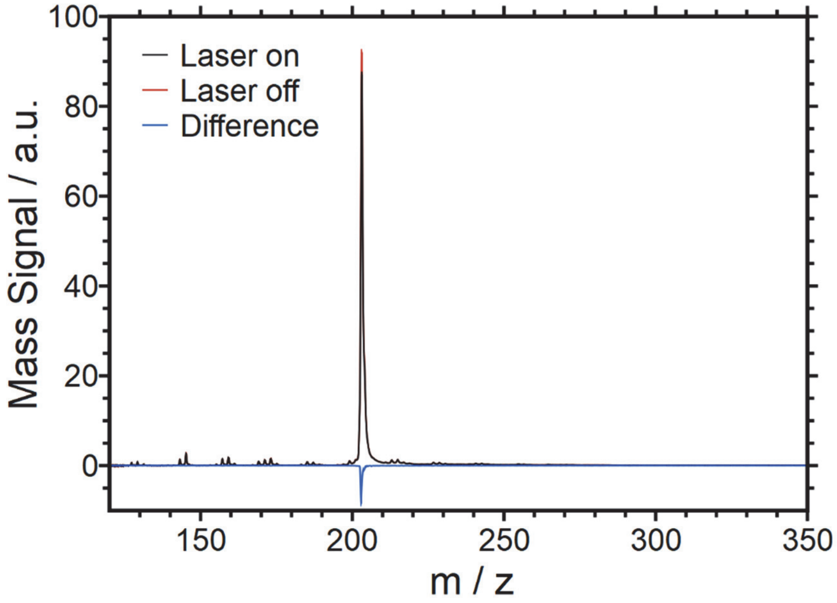

Figure S 2: Mass Spectrum of Trp. Blue: Difference induced by the laser pulse, highlighting the photodetachment of the Trp-monomer anion.

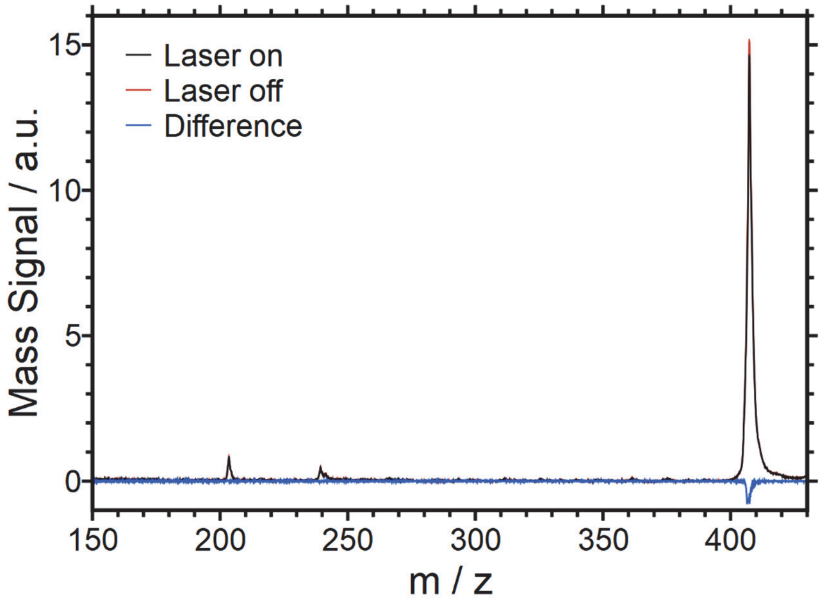

Figure S 3: Mass Spectrum of Trp. Blue: Difference induced by the laser pulse, highlighting the photodetachment of the Trp-dimer anion.

Figure S 4 shows the raw data of electron time-of-flight spectra employed for the calculation of the PECD. For the evaluation of the PECD just the main signal marked by grey vertical lines is used. The smaller signal at later times has, up until now, not shown to hold useful information.

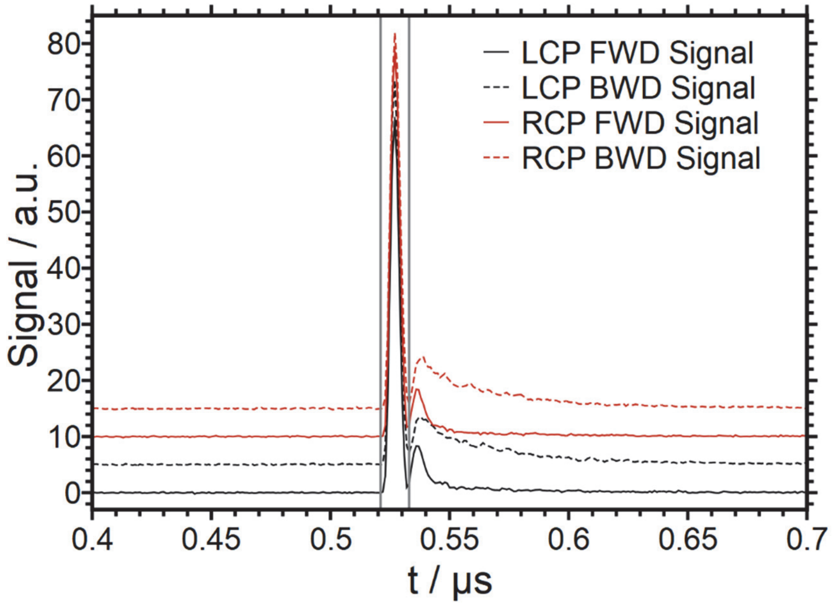

Figure S 4: Example Time-of-Flight of electrons used for the PECD measurement. Depicted is a D-Phe measurement, each line represents an average of 300 laser shots for noise reduction. The traces were stacked in the graph to make a distinction possible. The gray lines depict the integration boundaries used for the PECD calculation.
